# Solid pseudopapillary neoplasms of the pancreas in childhood and adolescence—an analysis of the German Registry for Rare Pediatric Tumors (STEP)

**DOI:** 10.1007/s00431-023-05203-w

**Published:** 2023-09-21

**Authors:** Christian Jentzsch, Jörg Fuchs, Abbas Agaimy, Christian Vokuhl, Gabriele Escherich, Claudia Blattmann, Steven W. Warmann, Andreas Schmidt, Jürgen Schäfer, Ines B. Brecht, Dominik T. Schneider, Michael Abele

**Affiliations:** 1 Pediatric Hematology/Oncology, Department of Pediatrics, University Hospital Tuebingen, Tuebingen, Germany; 2 Pediatric Surgery and Pediatric Urology, Department of Pediatrics, University Hospital Tuebingen, Tuebingen, Germany; 3 Institute of Pathology, University Hospital Erlangen, Erlangen, Germany; 4 Section of Pediatric Pathology, Department of Pathology, University Hospital Bonn, Bonn, Germany; 5 Department of Pediatric Hematology and Oncology, University Medical Center Hamburg-Eppendorf, Hamburg, Germany; 6 Department of Pediatric Oncology, Hematology and Immunology, Olgahospital, Klinikum Stuttgart, Stuttgart, Germany; 7 Section of Pediatric Radiology, Department of Diagnostic and Interventional Radiology, University Hospital Tuebingen, Tuebingen, Germany; 8 Clinic of Pediatrics, Klinikum Dortmund, University Witten/Herdecke, Dortmund, Germany

**Keywords:** Solid pseudopapillary neoplasm, SPN, Children, Rare tumor, STEP

## Abstract

Solid pseudopapillary neoplasms (SPNs) are the most common entity among pediatric pancreatic tumors. Still, these are rare tumors with an annual incidence of 0.1–0.2/1,000,000, and little is known about their optimal treatment. This analysis aimed to increase knowledge about the occurrence and treatment strategies of SPN in childhood. Data regarding diagnostics, treatment, and outcome of children aged 0–18 years with SPN recorded in the German Registry for Rare Pediatric Tumors (STEP) were analyzed. Thirty-eight patients were identified with a median age of 14.5 years at diagnosis (range: 8–18) and a female preponderance (81.6%). The most frequent location of the tumor was the pancreatic tail. In histopathological and immunohistochemical examination, pseudopapillary, solid, and cystic lesions as well as expression of beta-catenin, progesterone receptors, and cyclin D1 were the most common findings. All patients underwent surgical resection. Most patients underwent open resection, predominantly tail resection for tumors in the tail region and pylorus-preserving pancreaticoduodenectomy for tumors in the head region. The main postoperative sequela was exogenous pancreatic insufficiency (23.7%), especially with SPN in the pancreatic head. No recurrence occurred during follow-up, although two patients underwent resection with microscopic residue.

*Conclusion*: SPN of the pancreas in childhood are low-grade malignancies with usually favorable treatment outcomes. However, therapy can lead to relevant long-term sequelae. To prevent recurrence, complete surgical resection is recommended, sparing as much healthy pancreatic tissue as possible. Interdisciplinary collaboration between specialists is essential to optimize treatment. Molecular genetic analysis of these tumors could improve understanding of their genesis.

**Table:** 

**What is Known:** *• Solid pseudopapillary neoplasms (SPNs) of the pancreas are very rare tumors in childhood.* *• Little is known about tumorigenesis, and there are no specific guidelines for treatment and follow-up in pediatric patients.*	
**What is New:** *• Characteristics, treatment, and outcome were comprehensively assessed in a large cohort of pediatric patients with SPN.* *• We propose recommendations for diagnosis, treatment, and follow-up of children with SPN, based on our analysis and considering published experience.*

**What is Known:**

*• Solid pseudopapillary neoplasms (SPNs) of the pancreas are very rare tumors in childhood.*

*• Little is known about tumorigenesis, and there are no specific guidelines for treatment and follow-up in pediatric patients.*

**What is New:**

*• Characteristics, treatment, and outcome were comprehensively assessed in a large cohort of pediatric patients with SPN.*

*• We propose recommendations for diagnosis, treatment, and follow-up of children with SPN, based on our analysis and considering published experience.*

## Introduction

Solid pseudopapillary neoplasm (SPN) of the pancreas is a low-grade malignant tumor with an annual incidence rate of 0.2/1,000,000 in childhood, which classifies them as very rare tumors [1–3]. In absolute numbers, most SPNs are diagnosed in adult patients, with a mean age at diagnosis of 30 years and a clear preponderance of women. They account for 2–3% of all pancreatic neoplasms in adulthood [4]. In contrast, despite their rare occurrence, SPNs are the most common pancreatic tumor in childhood (61%), ahead of pancreatoblastoma (PB), pancreatic neuroendocrine tumors, and carcinomas [5]. In general, SPNs have a good prognosis. However, significant morbidity may develop postoperatively, and a low but existing tumor-related mortality of 1–2% has been reported [5]. Therefore, the establishment of standardized treatment concepts is urgently required in order to lower the therapeutic burden and the risk of sequelae. In order to improve existing approaches in the diagnosis and therapy of SPN, we analyzed pediatric patients with SPN enrolled in the German Registry for Rare Pediatric Tumors (STEP) with special emphasis on diagnostics, treatment, complications, and outcome.

## Materials and methods

This retrospective analysis was based on all patients with a diagnosis of SPN enrolled in the database of the STEP registry between 2008 and 2022. The STEP registry is a prospective hospital-based clinical registry of the German Society for Pediatric Oncology and Hematology (GPOH), specifically designed for children and adolescents with very rare tumors. Included are children and adolescents ≤ 18 years of age who met diagnostic criteria as per the local pathologist’s diagnosis. Pseudonymized data for patient-, tumor-, and treatment-related characteristics were collected. Frequencies refer to cases with recorded data. The staging of SPN is analogous to that of exocrine pancreatic carcinoma and was based on the TNM classification of the Union for International Cancer Control (UICC) [6]. All patients and/or their legal guardians gave informed consent for data collection and analysis at the time of diagnosis. The STEP registry was approved by the institutional review boards of the University of Erlangen (Re. No. 4340), the University of Tuebingen (Re. No. 847/2019BO2), and the ethics committees of the participating hospitals.

## Results

Thirty-eight pediatric patients with SPN were included in the STEP registry, diagnosed between 2008 and 2022 (Table 1). Thirty-one of them were female (81.6%), and seven were male (18.4%). The median age at diagnosis was 14.5 years. Further summarized characteristics of these patients are shown in Table 1. Detailed information on the individual patients is presented in Table 2.

**Table 1:** Patient characteristics aged 0–18 years registered in the German Registry for Rare Pediatric Tumors (STEP) with diagnosis of solid pseudopapillary neoplasms (SPNs) of the pancreas

**Characteristics**	**Count (frequency)/median (interquartile range, IQR)**
**Total count**	38 (100%)
**Sex**	
Male	7 (18.4%)
Female	31 (81.6%)
**Localization of the tumor**	
Head	14 (36.8%)
Corpus	3 (7.9%)
Tail	19 (50%)
Unspecified	2 (5.3%)
**Median age at diagnosis (years)**	14.5 (IQR 13–15.3)
**Median size of the tumor (cm)**	8 (IQR 5.3–9.65)

**Table 2:** Tumor characteristics in patients aged 0–18 years registered in the German Registry for Rare Pediatric Tumors (STEP) with diagnosis of solid pseudopapillary neoplasms (SPNs) of the pancreas

**Patient**	**Sex**	**Age in years**	**Localization**	**TNM classification**	**Primary and secondary diagnostics**	**Mode of surgery**	**Additional surgery**	**Resection status**	**Complication and sequelae following surgery**	**Status (follow-up-duration in months)**
1	F	15	Tail	T3, N0, M0	Sonography, MRI	Resection of the tail	None	R_0_	No	CCR (12)
2	F	15	Tail	T3, N0, M0	Sonography, CT	Resection of the tail	Partial splenectomy	R_0_	Pancreatic pseudocyst	CCR (2)
3	F	8	Tail	T1, N0, M0	Sonography, MRI	Enucleation	Appendectomy	R_0_	No	CCR (74)
4	M	14	Tail	T1, N0, M0	Sonography, MRI	Resection of the tail	None	R_0_	No	CCR (39)
5	F	16	Tail	T3, N0, M0	Sonography, MRI	Extirpation of the tumor	None	R_0_	Pleural effusion, pneumothorax, pancreatitis, splenic infarction, portal vein thrombosis	CCR (25)
6	M	15	Tail	T3, N0, M0	Sonography, MRI	Resection of the tail	Splenectomy	R_0_	No	CCR (90)
7	F	15	Tail	T3, N0, M0	MRI, -	Subtotal Resection of the tail	None	R_0_	Exocrine insufficiency of the pancreas	CCR (53)
8	F	14	Tail	T3a, N0, M0	Sonography, MRI	Resection of the corpus and the tail	None	R_1_	Varices of the fundus due to obliteration of the Vena lienalis	CCR (24)
9	F	18	Tail	T3, N0, M0	Sonography, -	Resection of the tail (by Laparoscopy)	None	R_0_	No	CCR (79)
10	F	12	Tail	T3, N0, M0	Sonography, MRI	Resection of the tail	Splenectomy, Resection of infiltrated peripancreatic soft tissue and omentum	R_0_	No	CCR (45)
11	F	16	Tail	T3, N0, M0	Sonography, -	Resection of the tail	None	R_0_	No	CCR (24)
12	F	11	Tail	T2, N0, M0	Sonography, MRI	Resection of the tail	None	R_0_	Diffuse venous rebleeding	CCR (34)
13	F	15	Tail	T3, N0, M0	CT, MRI	Resection of the tail (DaVinci^®^)	Splenectomy	R_0_	No	CCR (1)
14	F	11	Tail	T3, N0, M0	Sonography, MRI	Resection of the tail	Splenectomy, Inguinal lymph node dissection	R_0_	No	CCR (14)
15	M	15	Tail	T3, N0, M0	unknown	Resection of the tail	Splenectomy	R_0_	No	CCR (6)
16	F	17	Tail	T3, N0, M0	Sonography, MRI	Resection of the tail	Splenectomy	R_0_	Ascites, infectious retention in the spleen lodge, bilateral pleural effusions	CCR (11)
17	F	15	Tail	T3, N0, M0	Sonography, MRI	Resection of the tail	None	R_0_	No	CCR (22)
18	F	13	Tail	T2, N0, M0	Sonography, MRI	Resection of the tail	None	R_0_	No	CCR (19)
19	F	15	Tail	T3, N0, M0	Sonography, MRI	Extirpation of the tumor	None	R_0_	No	CCR (21)
20	F	13	Head	T3, N0, M0	Sonography, MRI	Enucleation	None	R_1_	No	Alive (unknown)
21	F	16	Head	T3, N0, M0	CT, MRI	PPPD	Cholecystectomy	R_0_	Exocrine insufficiency of the pancreas	CCR (38)
22	F	16	Head	T3, N0, MX	Unknown	Whipple	None	R_0_	No	CCR (72)
23	F	14	Head	T3, N0, M0	Sonography, MRI	PPPD	Cholecystectomy, Resection superior mesenteric vein	R_0_	Exocrine insufficiency of the pancreas, secondary amenorrhea	CCR (27)
24	F	12	Head	T3, N0, M0	Sonography, MRI	PPPD	None	R_0_	No	CCR (47)
25	F	17	Head	T3, N0, M0	Sonography, MRI	PPPD	None	unknown	Exocrine insufficiency of the pancreas	Alive (unknown)
26	F	16	Head	T3, N0, M0	Sonography, MRI	Partial resection of the pancreatic head	None	R_0_	Pleural effusion	CCR (23)
27	M	14	Head	T2, N0, M0	MRCP, -	PPPD	Cholecystectomy	R_0_	Unknown	CCR (28)
28	F	15	Head	T2, N0, M0	Sonography, MRI	Whipple	Cholecystectomy	R_0_	Intermittent vomiting after food intake	CCR (4)
29	F	13	Head	T3, N0, M0	Sonography, MRI	PPPD	None	R_0_	Temporary exocrine insufficiency of the pancreas (< 7 months)	CCR (8)
30	F	14	Head	T3, N0, M0	Sonography, MRI	Whipple	None	R_0_	Exocrine insufficiency of the pancreas, bilateral pleura effusion	CCR (4)
31	M	10	Head	T2, N0, M0	MRI	DPPHR	None	R_0_	Exocrine insufficiency of the pancreas	CCR (11)
32	F	13	Head	T2, N0, M0	Sonography, MRI	DPPHR (by laparoscopy)	None	R_0_	Exocrine insufficiency of the pancreas; PTBD due to obstruction of choledochal duct	CCR (77)
33	F	15	Head	T3, N0, M0	Sonography, MRI	DPPHR	None	R_0_	Exocrine insufficiency of the pancreas	CCR (15)
34	M	14	Corpus	T3, NX, M0	Sonography, MRI	Pancreatic segment resection	None	R_0_	Pleural effusion	CCR (5)
35	F	13	Corpus	T3, N0, M0	Sonography, MRI	Resection of the corpus and tail	None	R_0_	No	CCR (25)
36	M	14	Corpus	T3, N0, M0	MRI, endosonography	Resection of the tail (DaVinci^®^)	Splenectomy, resection of the left colon flexure	R_0_	Unknown	Alive (unknown)
37	F	11	Between Corpus and Tail	T3a, N0, M0	Sonography, -	Whipple	None	R_0_	Unknown	CCR (9)
38	F	16	Between Corpus and Tail	T3, N0, M0	Sonography	Resection of the tail (by laparoscopy)	None	R_0_	unknown	CCR (19)

*F* female, *m* male, *MRI* magnetic resonance imaging, *CT* computed tomography, *MRCP* magnetic resonance cholangiopancreatography, *PPPD* pylorus-preserving pancreaticoduodenectomy, *DPPHR* duodenum-preserving pancreatic head resection, *R*_0_ resection without microscopic residue, *R*_1_ resection with microscopic residue, *PTBD* percutaneous transhepatic biliary drainage, *CCR* complete clinical remission

Most frequently, patients presented with abdominal pain (*n* = 20), nausea (*n* = 8), vomiting (*n* = 6), and palpable mass (*n* = 7). In addition to these more frequent complaints, other symptoms occurred in individual cases (e.g., diarrhea, flank pain, bloating, *n* = 2 each). The duration of presenting symptoms prior to diagnosis was mostly 1–2 months (range: 2 days–9 months). In eight patients, the diagnosis of SPN was an incidental finding. This included incidental findings after trauma (e.g., blunt abdominal trauma in sport; *n* = 5) as well as routine examinations due to other reasons. Obesity (*n* = 6) and overweight (*n* = 2) were frequent concomitant diagnoses. None of the patients presented with jaundice.

The suspected diagnosis was initially made by sonography (*n* = 30) of an unclear mass (*n* = 26), which was then further investigated by magnetic resonance imaging (MRI; *n* = 26). Other suspected diagnoses included tumors of the left kidney (*n* = 3) or neuroblastoma (*n* = 1). The definitive diagnosis was made by a pathological examination of the resected tumor (*n* = 25) or by endosonography-guided biopsy (*n* = 3) or, in individual cases, by sonography-guided punch biopsy, MRI- or computed tomography (CT)-guided biopsies, and endoscopic biopsy (*n* = 1 each; six patients without clear indication whether the diagnosis was based on tissue from biopsy or resection). Additional pathologic examination by a reference pathologist was performed in 24 cases confirming the diagnosis. Alpha-fetoprotein (AFP) was not elevated in any patient at the time of diagnosis.

All patients underwent surgical resection of the SPN. An open surgical approach was chosen most frequently (*n* = 33), only three patients were treated by laparoscopy. In two other patients, the tumor was resected in a robotic-assisted surgery using the DaVinci^®^ surgical system.

When the tumor was localized in the tail of the pancreas (*n* = 19), resection of the tail was the method of choice (*n* = 15), followed by extirpation (*n* = 2), and enucleation (*n* = 1) of the tumor and combined resection of tail and corpus in one patient. The spleen was removed simultaneously in seven patients. Lymph node dissection was performed in two patients without evidence of lymph node metastases. The tumor in one patient showed extension beyond the pancreas with infiltration of the peripancreatic tissue including the greater omentum. Therefore, these anatomical structures were resected additionally. Except for one patient, all patients achieved resection with free margins (*R*_0_; *n* = 18). In one patient, residual cancer cells were identified microscopically at the margin (*R*_1_), but the patient remained in remission during a follow-up of 24 months.

For tumors localized to the head of the pancreas (*n* = 14), pylorus-preserving pancreaticoduodenectomy (PPPD) prevailed as the surgical technique (*n* = 6), followed by duodenum-preserving pancreatic head resection (*n* = 3) and the Whipple procedure (pancreaticoduodenectomy with removal of the gallbladder and occasionally part of the stomach; *n* = 3). In one patient each, the tumor was enucleated and a part of the pancreatic head was resected. In 4 patients, cholecystectomy was additionally performed. Twelve patients achieved *R*_0_, one patient had a *R*_1_ resection. However, no recurrence occurred in this patient during a follow-up of 3 years. In one patient, information regarding the R-status was missing.

The rare localizations in the corpus (*n* = 3) and between the corpus and tail of the pancreas (*n* = 2) were treated with different individualized resection procedures. Pancreatic segment resection (*n* = 1), tail resection (*n* = 2), Whipple procedure (*n* = 1), and simultaneous resection of the tail and corpus (*n* = 1) were performed. All of these patients remained recurrence-free during follow-up.

As vital pancreatic tissue was removed or displaced by the treatment or tumor, special attention was paid to postoperative morbidity. Exogenous pancreatic insufficiency was one of the most relevant sequelae. A distinction was made between temporary insufficiency (*n* = 1) and permanent insufficiency (*n* = 8). Patients with permanent insufficiency were treated continuously with pancreatic enzyme replacement therapy, whereas the patient with temporary insufficiency only needed such treatment for less than seven months after surgery. Postoperative complications were pleural effusions (*n* = 5), ascites (*n* = 2), pancreatitis (*n* = 1), varices of the fundus (*n* = 1), splenic retention (*n* = 1), pancreatic pseudocyst (*n* = 1), and diffuse venous bleeding (*n* = 1). Seven patients received antibiotic infection prophylaxis for different durations after splenectomy, depending on their age, to prevent an overwhelming post-splenectomy infection. Apart from the patients with pancreatic insufficiency requiring treatment, no other long-term sequelae were reported during the follow-up.

Relevant microscopic and immunohistochemical findings are summarized in Table 3. On histopathologic examination, pseudopapillary (*n* = 34, 89.5%) and solid (*n* = 31, 81.6%) patterns were found most frequently. In addition, hemorrhages (*n* = 18; 47.4%) as well as cystic (*n* = 18, 47.4%) and fibrosclerotic (*n* = 9, 23.7%) areas were frequently encountered. A tumor capsule was found in 17 patients (44.7%). Immunohistochemically, aberrant nuclear beta-catenin expression as a defining feature was detected (35/36 positive) followed by the nuclear progesterone receptor (28/30 positive). Cyclin D1 (27/28 positive), vimentin (23/23 positive), CD10 (23/23 positive), CD56 (18/18 positive), and alpha-1-antitrypsin (15/16 positive) were also among the more frequent findings.

**Table 3:** Microscopical and immunohistochemical findings in patients aged 0–18 years registered in the German Registry for Rare Pediatric Tumors (STEP) with diagnosis of solid pseudopapillary neoplasms (SPNs) of the pancreas

**Parameter**	**Count (frequency)**
**Microscopical findings**	
Pseudopapillary	34/38 (89.5%)
Solid	31/38 (81.6%)
Cystic	18/38 (47.4%)
Hemorrhages	18/38 (47.4%)
Tumor capsule	17/38 (44.7%)
Fibrotic	9/38 (23.7%)
Hyaluronic	8/38 (21.1%)
Necrotic	7/38 (18.4%)
Rosettes	5/38 (13.2%)
**Immunohistochemistry**	
Beta-catenin	n = 35 positive, n = 1 negative, n = 2 N.D.
Progesterone-receptor	n = 28 positive, n = 2 negative, n = 8 N.D.
Cyclin D1	n = 27 positive, n = 1 negative, n = 10 N.D.
Vimentin	n = 23 positive, n = 0 negative, n = 15 N.D.
CD10	n = 23 positive, n = 0 negative, n = 15 N.D.
Synaptophysin	n = 20 positive, n = 9 negative, n = 9 N.D.
CD56	n = 18 positive, n = 0 negative, n = 20 N.D.
Alpha-1-antitrypsin	n = 15 positive, n = 1 negative, n = 22 N.D.
Pan-cytokeratin	n = 15 positive, n = 2 negative, n = 21 N.D.
Neuron specific enolase	n = 10 positive, n = 2 negative, n = 26 N.D.
**Molecular findings**	
Patient 6	*CTNNB1* Exon 3, type p.S37F
Patient 8	*CTNNB1* Exon 3, type p.G43R, c.100G>A
Patient 14	*CTNNB1* Exon 3, type p.D32N, c.94G>A
Patient 15	*CTNNB1* Exon 3, type p.G43R
Patient 23	*CTNNB1* Exon 3, type p.Asp32Tyr, c.94G>T
	*FGFR1* Exon14, type 9.Ile639Met, c.1917A>G
Patient 28	No pathogenic findings

*N.D.*  not determined

In six patients, molecular genetic data were available. Five patients were found to have a genetic variant with a single-nucleotide base change and subsequent amplification in *CTNNB1* exon 3. The alterations clustered in the critical regions of codons 32 and 43. In one patient, no *CTNNB1* variant or other pathogenic alteration could be found. Another alteration was detected in one patient at *FGFR1* exon 14.

After surgical resection, all patients underwent follow-up. No relapse of the disease was observed in any patient. The median follow-up period was 23 months (IQR 11–39).

## Discussion

In this retrospective analysis of 38 STEP patients with pancreatic SPN, we found a significant preponderance of females, consistent with previous reports [5, 7–9]. An association with female sex hormones in adolescents of childbearing age and young adults has been suggested as potential explanation [10]. Whether obesity and overweight, which appeared to be disproportionately prevalent in our patient cohort, are linked to this, requires further investigation [11]. Studies in young adults showed that progesterone may act as an oncogenic factor [10, 12]. Since SPN lack pancreas-specific transcription factors and show accumulation of beta-catenin, an origin from embryonic stem cells of the genital ridge has been discussed, which is supported by the frequent expression of progesterone receptors in our series [13, 14]. However, the expression of neuroendocrine markers may argue for an origin from pluripotent stem cells of the pancreas supporting other reports [14, 15]. Other than representing a form of “Wnt-opathy”, the exact etiology of SPN remains unclear, similar to other organ-specific entities that share beta-catenin alteration with impaired Wnt signaling and frequent expression of progesterone receptor and CD10 [16].

Since most SPN occurred in adolescent patients, age may serve as a distinguishing feature in children and adolescents with neoplasms of the pancreas, as PBs are more likely to occur in younger children [17]. As SPN very rarely develop distant metastases, primarily local, non-specific symptoms occur. In some cases, this can lead to a significant latency period during which the tumor disease can progress.

Sonography is the diagnostic method of choice for the evaluation of patients with unspecific abdominal symptoms or a suspected pancreatic mass [18]. For diagnostic confirmation, MRI of the abdomen was commonly used in our patients to characterize the tumor in detail and to allow for preoperative planning [19, 20]. Since MRI features can be highly suggestive for the diagnosis of SPN, we consider it useful for preoperative distinction of more aggressive pancreatic neoplasms [9]. In individual cases, CT imaging can provide additional information, e.g., about hemorrhages or vascular invasion [19, 20]. For a possible differentiation from PB, the AFP level should be determined at the time of diagnosis.

Biopsies were performed in only seven patients prior to resection because SPNs, unlike PB or ACC, have distinct features on imaging, avoiding further interventions other than curative surgery. Solid and cystic tumor segments, tumor hemorrhages, and tumor capsules may be indicative of SPN on imaging [19–21]. Conversely, deviating image morphological features may suggest the need for a biopsy in view of the different approach in other malignant neoplasms [18, 22].

Histopathological and immunohistochemical characterizations are essential, and assessment by an experienced reference pathologist should be sought. Pseudopapillary, mixed solid, and cystic lesions as well as fibrotic and hyalinized portions are indicative of SPN. Solid portions in larger tumors are thought to be associated with a higher grade of malignancy [23]. We demonstrated that the immunohistochemical detection of nuclear beta-catenin associated with the altered *CTNNB1* gene is most important, followed by progesterone receptors, vimentin, CD56, and CD10 [10, 24]. However, nuclear staining of β-catenin may also be seen in PB (limited to the squamous morules) and even in ACC and is therefore not sufficient as a distinguishing characteristic alone, so that the morphological and clinical context should be considered when interpreting the immunohistochemical findings [25]. We confirmed cyclin D1 and alpha-1-antitrypsin as further potential markers [16]. While synaptophysin may be positive, especially in differentiating neuroendocrine tumors, chromogranin A (*n* = 2) is usually negative in SPN [24, 26]. A more diffuse and strong pan-cytokeratin expression should alert to the possibility of a neuroendocrine neoplasm (NET) [16].

Molecular genetic analyses of tumor tissue were performed in six patients to detect driver alterations. The most frequently involved gene was *CTNNB1*, in which exon 3 was particularly affected, leading to nuclear accumulation of beta-catenin [27]. This is consistent with other studies conducted mainly in adult patients, in which *CTNNB1* was altered in over 90% of tumor samples [27, 28]. However, *CTNNB1* alterations have also been described in PB and ACC, ruling out an SPN-specific modification [29, 30]. In addition, one patient in our cohort had a sequence variant in the *FGFR1* gene, which has not previously been associated with SPN. In other tumor entities such as non-small cell lung cancer or breast cancer, *FGFR1* is frequently altered and a therapeutic target. Genetic analyses of tumor and healthy tissue are of particular scientific interest in order to further clarify the pathogenesis and etiology of SPN.

Resection of the pancreatic tail was performed in most of our patients as the tail was the most common tumor site (50%), in contrast to other studies where tumors were more often located in the pancreatic head [5, 7]. Individual surgical planning should consider laparoscopic or robotic-assisted spleen-preserving distal pancreatectomy, which can minimize postoperative complications [31, 32]. While Fais and colleagues found an increased risk of recurrence after laparoscopic biopsy and subsequent resection, no recurrences were noted in our patients after laparoscopic resection, consistent with other studies, while patients benefited from shorter hospitalization [33, 34]. Therefore, laparoscopic resection of SPN may be considered by experienced pediatric surgeons based on an individual risk assessment. In distal pancreatectomy, a substantial portion of the pancreas is resected, which resulted in exocrine pancreatic insufficiency in one patient in our cohort. In adults, the frequency of exocrine pancreatic insufficiency and diabetes mellitus has been reported in up to 17% and 23% of patients, respectively [35]. If concurrent splenectomy is required, appropriate asplenia treatment must be administered postoperatively to avoid overwhelming post-splenectomy infections, including relevant vaccinations.

For tumors localized in the pancreatic head, PPPD was performed more frequently than Whipple procedure in our cohort. The surgical approach to these tumors is controversial in the literature, mainly because of postoperative morbidities, but there are few data on pediatric patients [5, 36]. While one report analyzing different pancreatic neoplasms in childhood found higher rates of exocrine insufficiency after PPPD, favoring Whipple surgery, others found no differences in this regard [5, 36, 37]. An alternative surgical approach for pancreatic head tumors is duodenum-preserving pancreatic head resection, which is less radical in its extent and is primarily intended for benign and low-grade malignant tumors [9, 38]. In terms of postoperative sequelae, this method seems to offer an advantage, also with regard to the occurrence of endocrine insufficiencies [5, 39]. Furthermore, it can be performed safely laparoscopically as in one patient of our cohort [40, 41]. Due to its potential advantages, it may be considered for tumors of the pancreatic head with radio-morphologic criteria typical of SPN. Eight cases of exocrine pancreatic insufficiency after resection of a tumor in the pancreatic head occurred in our cohort (57.1%, thereof 7.1% with temporary enzyme replacement therapy for less than 7 months). Since this is an expected sequela after resection of the pancreatic head, the question arises whether the proportion of exocrine pancreatic insufficiencies could be even higher and corresponding information was missing in the follow-up reports. Pancreatic enzyme replacement therapy is essential in such cases. Endocrine pancreatic insufficiency (diabetes mellitus) did not occur in our cohort, but has been rarely reported after pancreaticoduodenectomy [42]. The varying frequencies of morbidities after surgery suggest a correlation with the surgeon’s experience; thus, resections of pancreatic tumors in children should only be performed by experienced pediatric surgeons.

To minimize the loss of pancreatic tissue, enucleation of the tumor is favored by some for small tumors, resulting in a lower probability of postoperative pancreatic insufficiency [43, 44]. However, due to an increased recurrence rate, enucleation should only be considered in selected cases [23, 45].

Apart from local extension beyond the pancreas with infiltration of adjacent anatomic structures, distant metastases and lymph node metastases are rare in SPN in the pediatric age group [46–48]. Infiltration of peripancreatic tissue and neural or vascular infiltration favor the development of metastases [49]. If metastases occur, these should also be radically removed, if possible, to prevent recurrence [50]. The data on additional cytostatic therapy are sparse. Among others, combinations of fluorouracil and oxaliplatin as well as etoposide, ifosfamide, and cisplatin have been applied in an adjuvant or neoadjuvant setting for recurrent peritoneal metastases [47, 51]. In individual cases, alternatives such as hyperthermic intraperitoneal chemotherapy or transarterial chemoembolization may also be considered [52, 53]. Especially in the case of local recurrence, these patients still have a good prognosis, although fatal courses have been described [5, 47, 48]. Overall, children and adolescents with SPN have an excellent prognosis with a survival rate of more than 95% [5, 47, 48].

In recent years, different criteria have been proposed that may correlate with more aggressive tumor biology, higher grade of malignancy, and higher recurrence probability: age at diagnosis <13.5 years, *R*_1_ resection, larger tumors and male sex [23, 47, 54]. Despite *R*_1_ resections in two cases, we did not detect any recurrences in our cohort. Thus, our data do not support the listed risk factors of recurrence as in other pediatric case series [23, 47, 48]. However, this should be viewed with caution, as recurrences in SPN have also occurred significantly later, in some cases up to 10 years after initial diagnosis [47, 55]. This highlights the need for long-term follow-up.

Currently, there are no standardized follow-up schedules. Based on our analysis and published experience, we propose follow-up appointments with clinical examinations and sonography of the abdomen every 3 months for the first 2 years after complete resection. After that, such follow-up should be performed every 6–12 months until 10 years after diagnosis, due to reports of late recurrences. In patients with *R*_1_ resections, the follow-up interval should be shortened to every 3 months, and alternating MRI and ultrasound should be considered for the first 2 years after resection. Thereafter, follow-up appointments with clinical examination and sonography of the abdomen should be performed every 6 months initially and then annually until 10 years after diagnosis.

## Conclusion

SPNs in children and adolescents are low-grade malignancies with an overall excellent oncological outcome. Nevertheless, unfavorable courses with aggressive tumor biology may rarely occur, and therapy may be associated with significant long-term sequelae. Recurrences can be prevented by complete resection, sparing as much healthy residual pancreatic tissue as possible to avoid exocrine and endocrine insufficiencies. Enucleation should be only be considered in selected cases as it may enhance intra-abdominal recurrences. Minimally invasive resection procedures can be evaluated, considering the respective tumor extent, but require sufficient expertise of the surgical oncologist. Close interdisciplinary collaboration is essential for optimal treatment of these patients. To clarify the pathogenesis of SPN in differentiation from other pancreatic malignancies, molecular genetic studies are of particular scientific interest. International collaborations such as the European Cooperative Study Group for Pediatric Rare Tumors (EXPeRT) are needed to improve our understanding of SPN in this age group by validating potential risk factors for recurrence in a larger prospective patient cohort with adequate long-term follow-up.

## Data Availability

The data that support the findings of this study are available on request from the corresponding author (M. A.). The data are not publicly available due to privacy restrictions.

## Abbreviations

ACC: Acinar cell carcinoma
AFP: Alpha-fetoprotein
CT: Computed tomography
EXPeRT: European Cooperative Study Group for Pediatric Rare Tumors
GPOH: German Society for Pediatric Oncology and Hematology
IQR: Interquartile range
MRI: Magnetic resonance imaging
PB: Pancreatoblastoma
PPPD: Pylorus-preserving pancreaticoduodenectomy
R_0_: Resection without microscopic residue
R_1_: Resection with microscopic residue
SPN: Solid pseudopapillary neoplasm of the pancreas
STEP: German Registry for Rare Pediatric Tumors
UICC: Union for International Cancer Control
